# Supplementary figures and images for: The Association of Cooking Fuel Use, Dietary Intake, and Blood Pressure among Rural Women in China
Source: Int J Environ Res Public Health. 2020 Jul 30;17(15):5516. doi: 10.3390/ijerph17155516 (PMC7432946; doi:10.3390/ijerph17155516)

**Figure S1: Summary of Participant Flow**

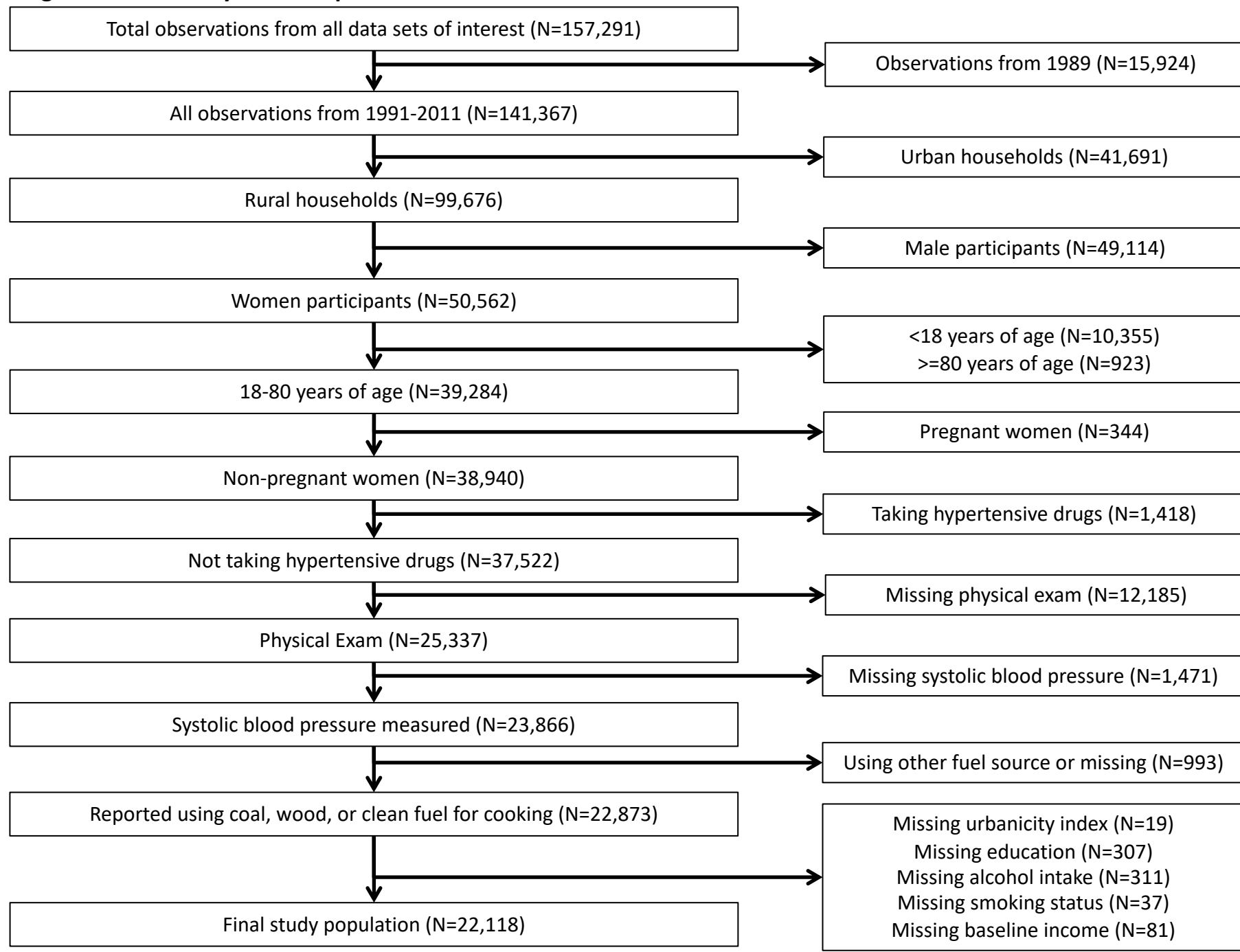

Supplement: Supplementary file 1 [file ijerph-17-05516-s001.pdf]
